# Domestic

**Published:** 1841-07-31

**Authors:** 


### DOMESTIC.

*Report of the Obstetric Practice in the Philadelphia Dispensary, for the Fourth, Fifth and Sixth months*, 1841. JOSEPH WARRINGTON, M. D., Accoucheur.

Twenty-five women have been delivered, at or near full term, of twenty-five children, of whom eighteen were boys, and seven girls.

The average duration of labour in twenty cases was thirteen and a half hours, the extremes being two and ninety-six hours.

The average time required for the spontaneous delivery of the placenta was, in nineteen cases, twenty minutes—the extremes being five and sixty minutes.

The fœtus presented the cephalic extremity in all cases. In nineteen cases in which the position of the fœtus was carefully noted, fifteen were in the first, two in the second, one in the the third, and one in the fifth of the vertex.

In one case the occiput presented originally transversely to the left ilium, in consequence of contraction of the sacro-pubal diameter of the superior strait. In this case the shoulders were delivered transversely at the inferior strait. The subject of this case was delivered by forceps, which could be made to lock only with the pivot directed toward the symphysis pubis. One blade, therefore, embraced the posterior portion of the left parietal bone, and the other passed over the right portion of the coronal suture and down upon the face, the head having rotated to correspond with the first position of the vertex—the impracticability of adjusting the forceps to that ascertained direction of the head, is ascribed to the arrangement of the superior strait. The child did well; the mother had a slight attack of metro-peritonites, but which was promptly subdued by free bleeding from the arm, and the application of leeches to the left iliac region.

Two of the children were still born; one apparently in a state of asphyxia, the labour having been so rapid, as to terminate before any assistance could be rendered; and the other in a semi-putrid state, the fœtus having evidently been dead some time before parturition.

One child was born in an extremely feeble state, after having been subjected to severe uterine contractions, several days successively, before the labour was terminated; it died in a state of exhaustion, four days after birth.

One child had erysipelas, commencing in the scalp, and spreading over the upper part of the body. It recovered under the persevering use of small doses of calomel, bicarbonate of soda, and powdered acacia internally, and the constant application of the mucilage of the slippery elm externally.

In one case the placenta was found to be adherent to the fundus of the uterus, requiring the careful use of the hand to separate it. Patient recovered without subsequent accident.

There were two cases in which the placenta was retained—one during five hours, and the other one hour and a half.

The placenta in both these cases, presented its whole internal disk to the os uteri, which was too small to transmit it. In one of these cases, also, the tonic contraction of the uterus was so feeble that ʒss. of ergot was given before it appeared expedient to use manual aid. In the other, also, the patient was suffering much from hæmorrhage; free frictions upon the abdomen, and the introduction of the hand into the uterus, were immediately sufficient. Both these cases subsequently did well.

In the subject of the delivery of the semi-putrid fœtus, though only nineteen years old, there was a large varicose tumour on the left labium. It rapidly subsided after delivery, with no other treatment than the free use of cold salt water.

Two of the cases which have been under care, required much attention for a long time before and after delivery. One had been the subject of inguinal abscess, supposed to have descended from the fascia of the psoas muscle, in whom the pressure of the gravid uterus was attended with great distress; although her labour was well conducted, she had an attack of peritonitis, which required great vigilance. She slowly recovered, without the recurrence of the abscess.

The other case was the subject of a scrofulous diathesis. She suffered during the latter months of pregnancy with severe pains over the whole abdomen, and especially in the right inguinal region. The labour was accomplished as in the other case, without extraordinary difficulty—but the patient passed through the various grades of peritonitis, metritis, and periostitis, and, finally, inguinal abscess. She at present offers some hope of recovery.

For most important aid, in the management of these protracted and critical cases, it is my duty to acknowledge the unremitting zeal of the members of my class, who participated in the treatment, and my thanks are due to the surgeons who gave us their council in the two cases last mentioned. Most of the physical comforts enjoyed by these poor women were furnished by the "Philadelphia Nurse Charity." There were several other cases of milder forms of peritonitis. They all recovered well under the usual treatment.

